# Response Properties of a Newly Identified Tristratified Narrow Field Amacrine Cell in the Mouse Retina

**DOI:** 10.1371/journal.pone.0137702

**Published:** 2015-09-09

**Authors:** G. S. Newkirk, M. Hoon, R. O. Wong, P. B. Detwiler

**Affiliations:** 1 Department of Physiology & Biophysics and Program in Neurobiology & Behavior, University of Washington, Seattle, Washington, United States of America; 2 Department of Biological Structure, University of Washington, Seattle, Washington, United States of America; University Zürich, SWITZERLAND

## Abstract

Amacrine cells were targeted for whole cell recording using two-photon fluorescence microscopy in a transgenic mouse line in which the promoter for dopamine receptor 2 drove expression of green fluorescent protein in a narrow field tristratified amacrine cell (TNAC) that had not been studied previously. Light evoked a multiphasic response that was the sum of hyperpolarizing and depolarization synaptic inputs consistent with distinct dendritic ramifications in the off and on sublamina of the inner plexiform layer. The amplitude and waveform of the response, which consisted of an initial brief hyperpolarization at light onset followed by recovery to a plateau potential close to dark resting potential and a hyperpolarizing response at the light offset varied little over an intensity range from 0.4 to ~10^6 Rh*/rod/s. This suggests that the cell functions as a differentiator that generates an output signal (a transient reduction in inhibitory input to downstream retina neurons) that is proportional to the derivative of light input independent of its intensity. The underlying circuitry appears to consist of rod and cone driven on and off bipolar cells that provide direct excitatory input to the cell as well as to GABAergic amacrine cells that are synaptically coupled to TNAC. Canonical reagents that blocked excitatory (glutamatergic) and inhibitory (GABA and glycine) synaptic transmission had effects on responses to scotopic stimuli consistent with the rod driven component of the proposed circuit. However, responses evoked by photopic stimuli were paradoxical and could not be interpreted on the basis of conventional thinking about the neuropharmacology of synaptic interactions in the retina.

## Introduction

Amacrine cells are the most diverse class of retinal neurons and the least understood [1]. There are approximately 40 different types—roughly 2 amacrines for each type of ganglion cell—but only a few have been studied in detail. This list would include the AII [2,3,4], A17 [5,6,7,8], starburst [9,10,11,12] and dopaminergic amacrine cells [11,13,14,15,16]. The principle reason for the lack of information about the different members of the amacrine cell family is the "needle in the haystack" problem. In order to properly study any chosen cell type it is first necessary to be able to find it reproducibly from one experiment to the next. The intact retina is an amorphous tissue that, with few exceptions, makes it impossible to accurately identify a particular cell type on what appears to be a homogeneous background sea of neurons. This problem may be surmounted, however, by using transgenic methods to selectively express fluorescent protein in specific retinal cells.

Here we report results obtained using two photon laser scanning fluorescence microscopy to make targeted whole cell recordings from a narrow field amacrine cell labeled by expression of fluorescent protein driven by the dopamine receptor 2 promoter in a BAC transgenic mouse from the Gensat retina project. This cell has not been studied previously making it the first of more than a dozen narrow field amacrine cell in the mammalian retina [17] to be characterized in terms of its morphology, light response properties and pharmacology.

## Materials and Methods

All experiments were performed in accordance with institutional and national guidelines for animal care approved by the Institutional Animal Care and Use Committee at the University of Washington. We used postnatal 21- to 50-day-old Gensat BAC transgenic mice (RP23-161H15) crossed into a C57/B6 background, where the GFP transgene was inserted following the ATG start codon of the *Drd2* promoter. All animals were housed in institutionally approved facilities at the University of Washington on a 12:12 hour light-dark cycle with *ad libitum* access to water and food.

### Tissue Preparation

Following 2 hours of dark adaptation, mice were killed in the dark using infrared illumination with image converters by cervical dislocation, and eyes were removed and placed in room temperature Ames’ medium (Sigma, St. Louis, MO) that was carbogenated (95% O_2_ and 5% CO_2_). The eyes were hemisected and the posterior half of the eyecup was bisected into equal pieces where the retina was isolated from the pieces as needed and adhered to a translucent Anodisc filter (Whatman, Florham Park, NJ) photoreceptor side down by wicking away excess solution. The retina and filter paper were transferred to a recording chamber fixed to the stage of a custom-built two-photon laser scanning fluorescence microscope, where the mounted retina was perfused with warmed (30–34°C) carbogenated Ames’ medium at a rate of 4–7 ml/min and viewed with a charge-coupled camera using infrared illumination.

### Cell Targeting

In the *Drd2*-*GFP* BAC transgenic mouse line, GFP expression was visualized in whole mount retina using two-photon microscopy as described previously [15,18,19,20]. Dual fluorescence excitation was achieved using a pump laser (Mira; Coherent) that delivered ~100-fs laser pulses of 930 nm light at 100 MHz with an average power of 4–8 mW measured at the front of the objective. Fluorescence emission was collected by a X40, 1.0-NA water-immersion objective (Nikon). Emission was split into two channels using custom band pass (BP) filters (Chroma Technology) to green (535 nm, BP 50 nm) and red (622 nm, BP 26 nm) fluorescence and collected by two independent photomultiplier tubes (Hamamatsu). The green channel was used to visualize GFP-positive cells in the inner nuclear layer (INL) and the red channel was used to visualize the recording pipette filled with an intracellular solution containing 80 μM Alexa Fluor 594 (Invitrogen). As previously reported, the *Drd2-GFP* BAC animal expresses GFP in two distinct populations of cells in the mouse retina [15]. TNAC cells were differentiated from dopaminergic amacrine cells on the basis of smaller soma size (7–9 μm to 14–16 μm diameter) and density (~500/mm^2^ compared to 50/mm^2^).

### Electrophysiology

To reach GFP-labeled cells in the INL, we used a pipette to micro-dissect a hole in the internal limiting membrane approximately 40–50 μm from the targeted cells [21]. Patch-clamp recordings were obtained using 5- to 7-MΩ electrodes, and signals were amplified using an Axopatch 200B amplifier (Axon Instruments). All recordings were made in current-clamp configurations using a standard internal solution containing (in mM) 122 K-gluconate, 10 Na-HEPES, 6 KCl, 6 K-EGTA, 3 Mg-ATP, and 0.2 Tris-GTP, brought to pH 7.4 with NaOH. Membrane voltages were corrected for a -11 mV liquid junction potential that was determined experimentally [22]. Voltage signals were filtered at 2 kHz and digitized at a sampling interval of 0.1 ms via an ITC-16 interface (Instrutech) using custom software written in Igor Pro (Wavemetrics) by Fred Rieke (University of Washington, Seattle, WA). Recording from the targeted GFP-expressing amacrine cell was confirmed by dye-filling with a red fluorescent dye (Alexa Fluor 594).

### Receptor Antagonists

To block specific synaptic transmissions, neurotransmitter receptor antagonists and agonists were added to the extracellular solution. These included (in μM): 40, GABAzine (SR-95531); 62.4, L-(+)-2-amino-4-phosphonobutyric acid (L-APB); 50, 2,3-dihydroxy-6-nitro-7-sulfamoyl-benzo[*f*]quinoxaline-2,3-dione (NBQX); 50, (1,2,5,6-tetrahydropyridin-4-yl) methylphosphinic acid hydrate (TPMPA); 2–10, strychnine; 50, D-(-)-2-Amino-5-phosphonopentanoic acid (AP5); 50, (*S*)-1-(2-Amino-2-carboxyethyl)-3-(2-carboxy-thiophene-3-yl-methyl)-5-methylpyrimidine-2,4-dione (UBP310) and 60, quinpirole. All chemicals were purchased from either Sigma or Tocris (Ellisville, MO).

### Light Stimuli

An optical bench [23] with a quartz-halogen light source was used to project light on to the photoreceptor layer of the retina through the sub-stage condenser. Unless specified otherwise, stimuli were 2-s steps of 720-μm- diameter (full field) circular spots of 440-nm light centered on the receptive field of the cell with an unattenuated intensity of 2.1 X 10^6^ photons μm^-2^ s^-1^, corresponding to 1.8 X 10^6^ light activated rhodopsin molecules (Rh*) rod^-1^ s^-1^ using a collecting area of 0.85 μm^2^ for mouse rods [24]. All experiments were performed with a 2 Rh*•rod^-1^•s^-1^ background intensity. To maintain the dark adaptational state of the retina the interval between sequential flashes was varied from 60 to 600 s, depending the intensity of the preceding stimulus.

### Immunohistochemistry

Retinas were isolated from eye-cups in cold oxygenated mouse artificial cerebrospinal fluid (mACSF, pH 7.4) containing (in mM): 119 NaCl, 2.5 KCl, 2.5 CaCl_2_, 1.3 MgCl_2_, 1 NaH_2_PO_4_, 11 glucose, and 20 HEPES, and mounted on membrane filters (Millipore, HABP013). Thereafter, retinas were immersion-fixed for 20 minutes in 4% paraformaldehyde in 0.1M phosphate buffered saline (PBS), rinsed, pre-incubated in PBS with 5% donkey serum and 0.5% triton (blocking solution) overnight, and then incubated in blocking solution for 3 nights at 4°C with primary antibodies. Antibodies used were directed against TH (mouse monoclonal, 1:500, Chemicon), GFP (rabbit polyclonal, 1:1000, Chemicon), and ChAT (goat polyclonal, 1:500, Chemicon). Secondary antibodies were anti-isotypic Alexa Fluor conjugates (1:1000, Invitrogen). Secondary antibody incubation was performed in PBS overnight, and the retinas mounted in Vectashield (Vector labs). For cell-fills, 5mM neurobiotin was used and retinas were post-fixed for 20 mins. To amplify the neurobiotin signal, streptavidin conjugated to Alexa Fluor 568 (1:200, Invitrogen) was used. Images were acquired on an Olympus FV1000 laser scanning confocal microscope, using a 1.35NA 60X Objective. Raw image stacks were processed using MetaMorph (Universal Imaging) and Amira (Mercury Computer Systems) software. Line scan profiles of fluorescence intensities were plotted using ImageJ (or Fiji).

## Results

### Somal distribution and morphology of a novel tristratified amacrine cell

**Fig 1:**
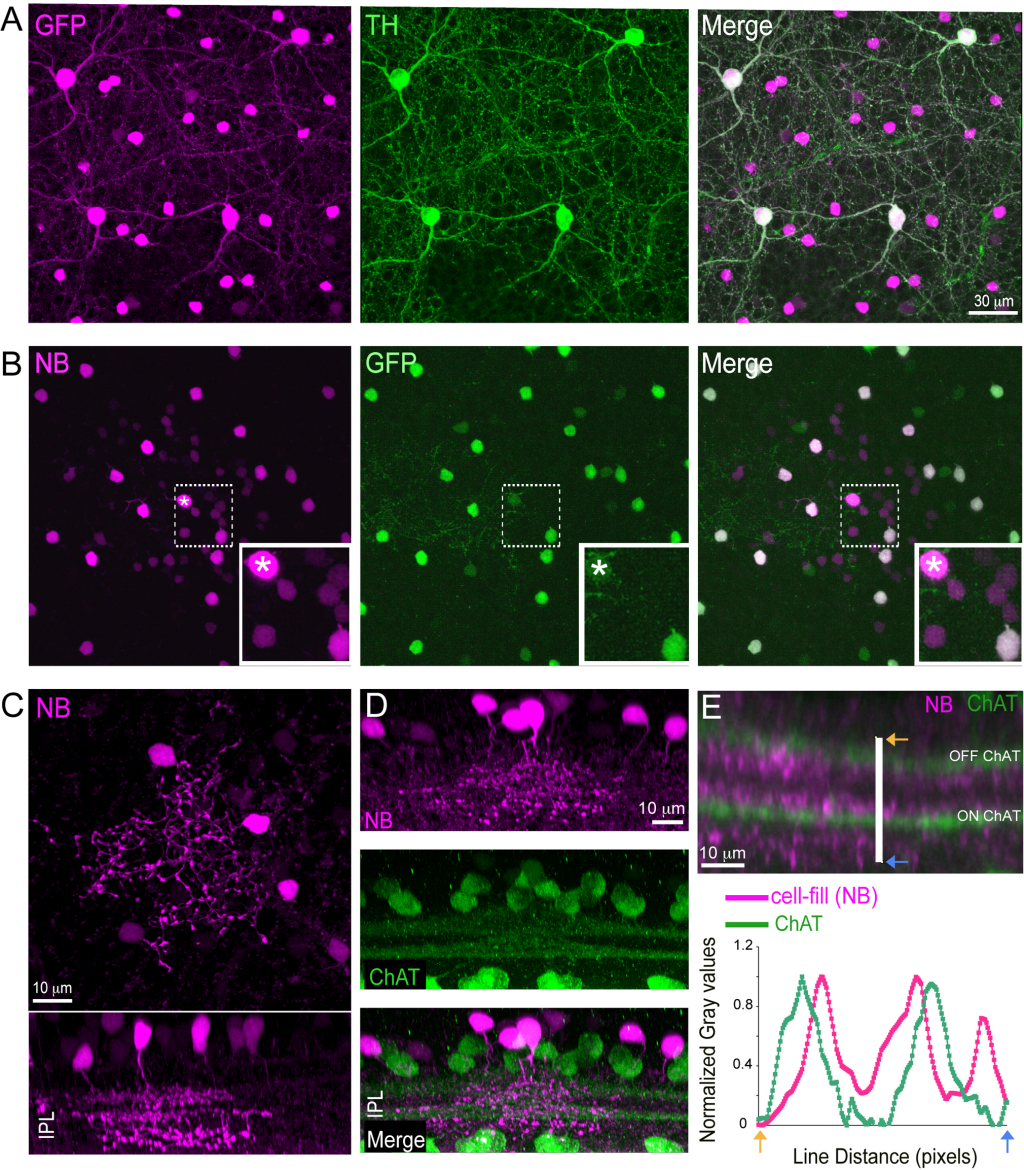
Morphology and lamination of a tri-stratified narrow-field amacrine cell. (**A**) Co-labeling with Tyrosine hydroxylase (TH, green) revealed two amacrine cell types in the *Drd2-GFP* (GFP, magenta) transgenic mouse retina. The smaller GFP positive cells are TH-negative. (**B**) Injecting Neurobiotin (NB, magenta) into an individual small-field GFP positive amacrine cell (marked with asterisk) revealed tracer-coupling to neighboring small somata that were also GFP positive (green) cells and to cluster of GFP negative cells with small somas in the retinal inner nuclear layer. Boxed regions enlarged in corresponding insets. (**C**) Neurite morphology of a Neurobioitin (NB, magenta) filled small GFP positive amacrine cell (top panel: *en face* view, bottom panel: side view). Side view reveals triple stratified neurites in the retinal inner plexiform layer (IPL). (**D**) Side view of Neurobiotin (NB, magenta) cell-filled tri-stratified amacrine cells in a retina co-labeled with choline acetyltransferase (ChAT, green). (**E**) Fluorescence intensity line scan (white line) showing the z- distribution of Neurobiotin filled processes and ChAT labeling across the retinal IPL (arrows mark the two ends of the line scan). The midpoint between the OFF and ON ChAT bands is approximately at the 50% IPL depth. Two ramification of the tri-stratified narrow-field amacrine cell processes lie between the ChAT bands with an additional profusion of processes that resides in the innermost lamina of the IPL, close to the ganglion cell layer.

In the GENSAT Drd2-GFP transgenic mouse fluorescent protein is selectively expressed in two different types of amacrine cells that can be distinguished on the basis of soma size, retina location and population density (Fig 1). The larger cells (14–17 μm diameter) are sparsely distributed(~ 25/ mm^2^) along the outer margin of inner plexiform layer (IPL). This, along with being stained by antibodies against tyrosine hydroxylase (Fig 1A), identifies the larger of the two cells as the dopaminergic amacrine cell [15]. The smaller cells, with soma diameters of ~ 7–9 μm, do not express tyrosine hydroxylase and are distributed in the inner most quarter of the inner nuclear layer (INL) at a uniform density (~ 500/mm^2^) across the retina [15].

The smaller cells were targeted for whole cell recording in whole mount retina using two photon laser scanning fluorescence microscopy [20]. Cells dialyzed with pipette filling solution containing neurobiotin were examined using confocal microscopy (see Methods) and found to be narrow field amacrine cells (Fig 1C). A single process extended from the cell body in the INL traveled vertically a short distance into the IPL before giving rise to three separate sets of lateral branches each extending over an area of 40–60 μm in diameter (Fig 1D). The plot of fluorescence intensity along a line traversing the IPL (Fig 1E) shows peaks in fluorescence corresponding to two separate dendritic ramifications located within the outer and inner bands of choline acetyltransferase (CHAT) (Fig 1D and 1E). A diffuse band of lobular endings in the innermost portion of the IPL, giving rise to a third peak in the line scan. Thus, the terminal arbors of this small-field amacrine cell type ramify in sublamina 2 of the OFF lamina, and at the border between sublamina 3 and 4, and within sublamina 5 of the ON sublamina. This amacrine cell has not been studied previously and will hence forth be referred to it as the tri-stratified narrow-field amacrine cell (TNAC). As discussed below, this cell has the same morphological features and stratification pattern as the only tristratified amacrine cell included in a recent connectomic analysis of the mouse retina, designated as AC42 [25].

### Dye coupling

When the expression of fluorescent protein was used to target a single cell for whole cell recording and the pipette filling solution contained neurobiotin we found that the dye had spread to stain a cluster of at least two different cell types. These included a group of approximately 10 GFP-positive cells that shared the same somal depth in the INL and dendritic stratification pattern as the targeted cell. Also trace-coupled to the injected TNAC were several more faintly stained cells that did not express GFP, with cell bodies that were located deeper in the INL than the soma of the targeted amacrine cell (Fig 1B). Since neurobiotin is a gap-junction permeant tracer [26] this suggests that TNACs are electrically coupled to each other as well as to an unidentified network of cells. Neither the extent of tracer coupling between TNACs nor the waveform or intensity dependence of their light response was altered by incubating the retina in extracellular solution containing 60 μM quinpirole, a selective dopamine receptor 2 agonist, for 60 min before beginning targeted TNAC recording in the same solution (data not shown).

### Physiological properties of TNACs

Whole cell current-clamp recordings were made from 48 TNACs in retinas from 31 DR2 transgenic mice. Resting membrane potentials in darkness, i.e. in the presence of a 2 Rh*/rod/s background light (see Methods), ranged between - 49 and - 70 mV in different cells (median -61 mV) with no evidence of spontaneous spike activity.

### Baseline noise

**Fig 2:**
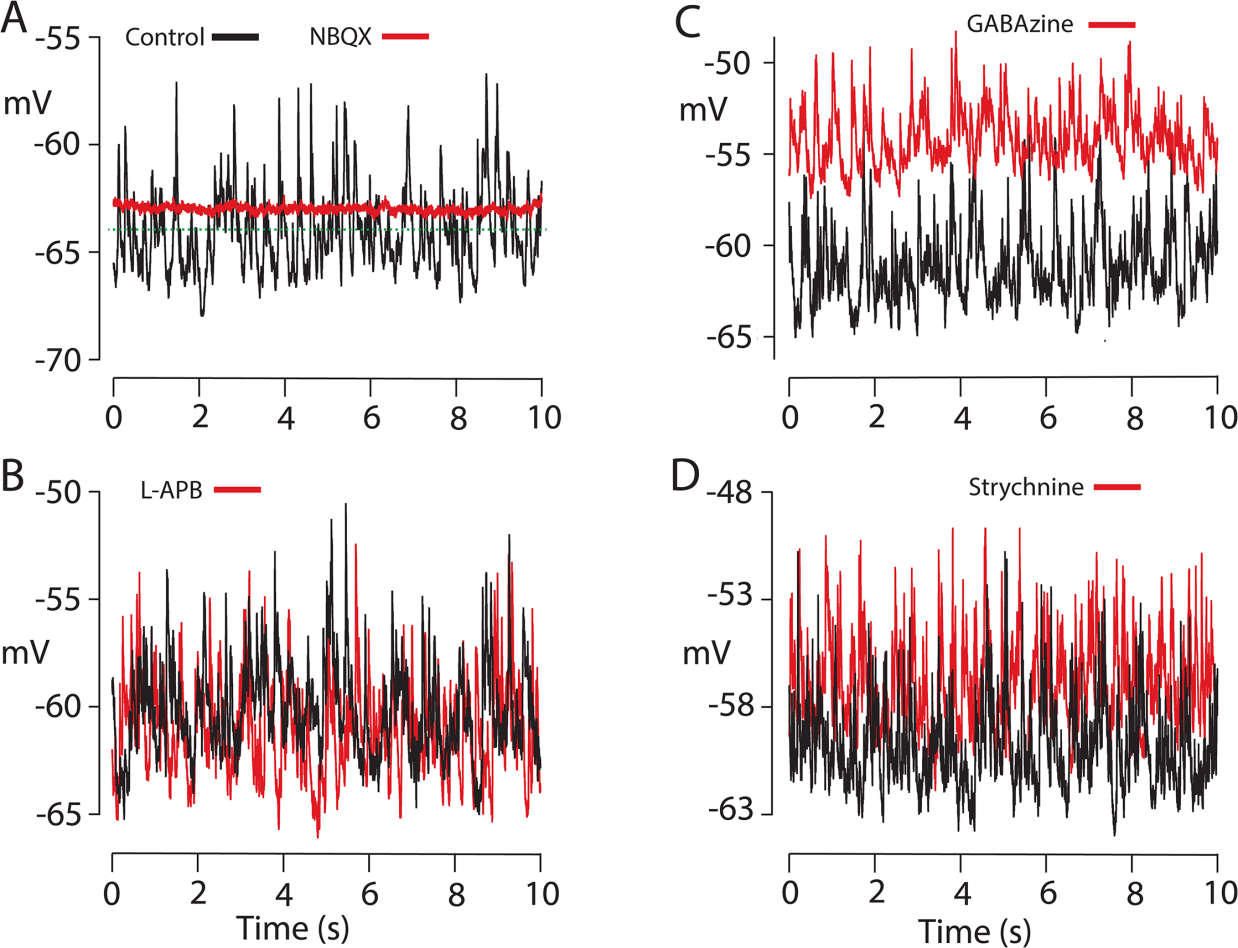
Spontaneous fluctuations in baseline voltage. Baseline noise recorded from single cells in darkness in absence and presence of: A. 50 μM NBQX; control (black): StDev 1.26 mV, maximum peak-to-peak (max p-to-p) amplitude 10 mV, mean V_rest_ (dashed line) -64 mV. NBQX (red): StDev. 0.11 mV, max p-to-p amplitude 1.2 mV, mean V_rest_ - 62.8 mV. Mean change V_rest_ caused by NBQX:+1.16 mV +/- 0.85 mV SEM, n = 6 (range = +4.8 to -1.0 mV). B. 62.5 μM L-APB; control (black): StDev 1.45 mV, max p-to-p 13 mV, mean V_rest_ -58.3 mV. L-APB (red): StDev 1.49 mV, max p-to-p amplitude 13.9 mV, mean V_rest_ -58.5 mV. The mean change V_rest_ caused by L-APB +0.08 mV (n = 5). C. 40 μM GABAzine; control (black): StDev 1.20 mV, max p-to-p amplitude 10 mV, mean V_rest_ -60 mV. GABAzine (red): StDev 1.26 mV, max p-to-p amplitude 11 mV, mean V_rest_ -51.8 mV. The addition of TPMPA to the GABAzine containing solution did not reduce the RMS (StDev) noise level compared to the level in control. Mean change V_rest_ was 6.25 +/- 1.10 mV n = 5 (range of change in Vrest 8.5 to 3 mV). Both values were significantly different than no change in voltage (p = < 0.003). D. 2 μM strychnine; control (black): StDev 1.45 mV, max p-to-p amplitude 14 mV, mean V_rest_ -56 mV. Strychnine (red): StDev 1.58 mV, max p-to-p amplitude 14 mV, mean V_rest_ -54 mV. Mean change V_rest_ caused by strychnine was +1.17 mV (n = 6), not significantly different than 0 change in voltage.

Recordings from TNACs were characteristically noisy with continuous fluctuations in dark resting potential with power distributed evenly between 1 to 20 Hz. In recordings from different cells the standard deviation (RMS) of the baseline dark noise (mean voltage set to 0 mV) ranged from 0.66 to 2.56 mV (mean 1.17 +/- 0.13 mV, n = 21) with maximum peak-to-peak amplitudes of 5 to 17 mV (mean 10.9 +/- 0.87 mV n = 21) The fluctuations in membrane potential persisted in the presence of inhibitory synaptic blockers (GABAzine, TPMPA and strychnine) and was blocked by NBQX with a small concomitant change in resting potential, mean 1.14 +/- 0.85 mV, n = 6, (Fig 2). Neither the baseline noise of the TNAC nor its resting membrane potential in darkness was affected by L-APB (Fig 2B). Whereas GABAzine, which had no effect of the RMS noise level (mean RMS before and after the addition of GABAzine were (n = 5): 1.19 +/- 0.24 mV versus 1.24 +/- 0.28 mV, respectively), caused a 3 to 9 mV (mean 6.25 +/- 1.1 mV, n = 5) depolarizing shift in the dark resting potential (Fig 2C). The addition of strychnine had no effect on TNAC resting potential or its baseline noise (Fig 2D).

### Light responses properties

**Fig 3:**
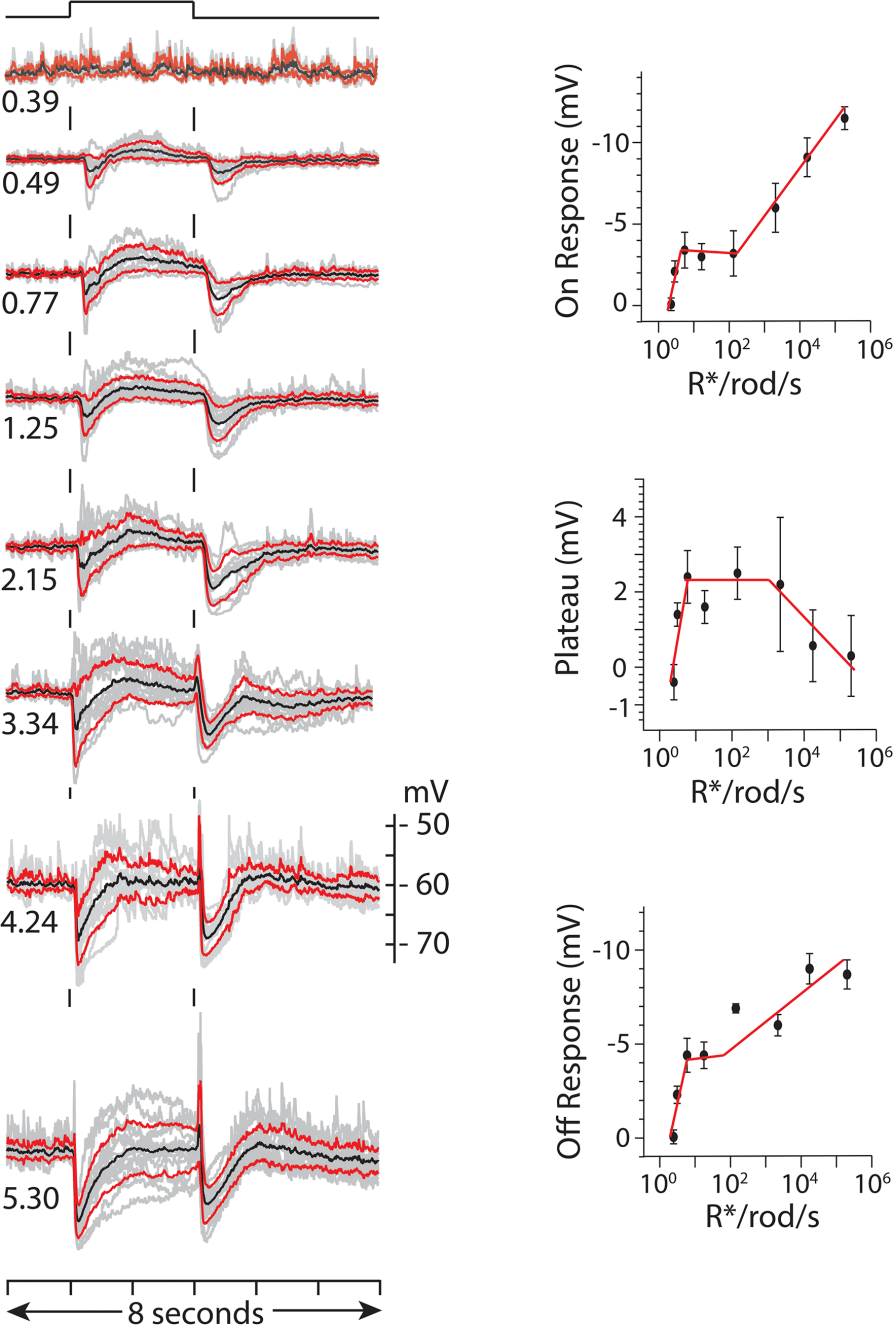
Response dependence on light intensity. Left column of traces show the collected average response (black), +/- standard deviation (red) and individual responses (gray) evoked by 2 s full field flashes (720 μm dia.) 440 nm light at indicated intensities (log_10_ Rh*/rod/s) recorded (current clamp) in different cells. The number of cells (n) that contributed to average response at each intensity (log_10_ R*/rod/s) were: n = 8 @ 0.39; n = 17 @ 0.49; n = 9 @ 0.77; n = 17, 1.25; n = 12 @ 2.15; n = 15 @ 3.34; n = 12 @ 4.24, n = 16 @ 5.30. Baseline voltage was set to the mean resting potential across all cells in the data set, i.e. -59.95 +/- 0.79 mV (+/- SEM, n = 21) range - 53 to -68 mV. The mean amplitude +/- SEM of the peak response within 400 ms of light onset, the response plateau (taken as mean voltage over 0.8 and 1.2 s of the 2 s step) and the peak hyperpolarizing response within 400 ms of light offset are plotted against light intensity in the three graphs to the right of the column of responses.

The average response evoked by 2 s exposure to full field (720 μm diameter spot) 440 nm light at intensities ranging from 0.39 to 5.95 log_10_ Rh*/rod/s can be described as consisting of three components: (1) an initial hyperpolarization at light onset that (2) recovered to a shoulder that plateaued to a level close (~ 1–2 mVs) to the dark resting potential and (3) a hyperpolarizing response at light offset (Fig 3, black traces). Responses evoked by a particular intensity were not, however, identical from one cell to the next (Fig 3, gray traces). In some cells the plateau phase of the flash response was reduced to such an extent that the response appeared to consist of only a short lived hyperpolarizing potential change at both light on- and offset. In other cells light onset evoked a transient depolarization that either cut short the initial hyperpolarizing phase of the ON response or preceded it. Similar variations in the waveform of the OFF response were also seen in different cells. The cell-to-cell differences in the shape of the light response were not associated with differences in resting potential, cell morphology or cell body location relative to inner margin of the INL.

The amplitudes of three phases of the average response varied with light intensity but only modestly with peak amplitudes between ~ +/- 10 mV of the resting potential (Fig 3, plots). The relationship between amplitude and stimulus intensity for all three components appeared to change course at intensities greater than 100 Rh*/rod/s when signals produced by cone input would be expected to begin to be recruited, at which point the amplitude of the ON and OFF responses increase linearly with the log_10_ of the light intensity. The intensity dependence of three response components was qualitatively the same for responses expressed as peak amplitude versus response area (data not shown).

There were no differences in the waveform of responses evoked by a small spot (35 μm diameter) centered on the cell and displaced from it by 650 μm. This suggests that over this expanse of retina the TNAC receptive field is not influenced by center-surround antagonism.

### Neuropharmacology

The three phases of the average light response suggests that it is the sum of a mixture of depolarizing and hyperpolarizing inputs that depend on their strength and temporal properties to give rise to responses that are generally similar but not necessarily identical from one cell to the next. To better identify the underlying synaptic inputs we examined the effects of neurotransmitter antagonists on the light response.

#### Effects of perturbing glutamatergic transmission on TNAC light response

**Fig 4:**
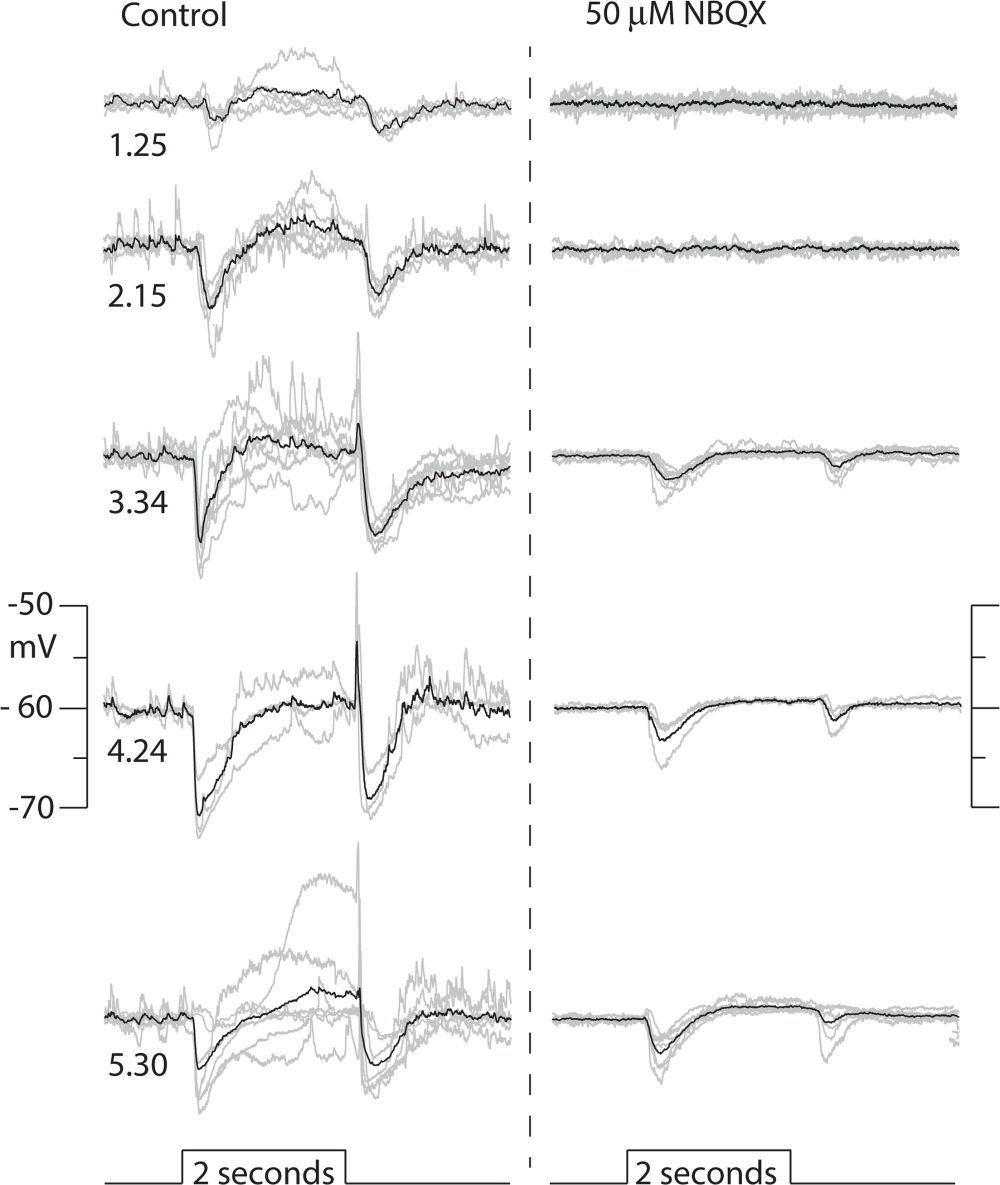
Effect of NBQX on responses to a range of light intensities. Right and left columns of traces show responses evoked by 2s flashes of full field 440nm light at the indicated intensities (log_10_ Rh*/rod/s) in the absence and presence of 50 μM NBQX. Each panel shows the individual responses recorded from n different cells (gray traces) and the their average (black). Resting potentials ranged across cells from -53 to -67 mV (mean -59 +/- 1.92, n = 7) and were set to - 60 mV for comparisons. The number of cells included in the average response at each intensity were: 1.25, n = 6; 2.15, n = 5; 3.34, n = 7; 4.24, n = 3; 5.40, n = 7.

**Fig 5:**
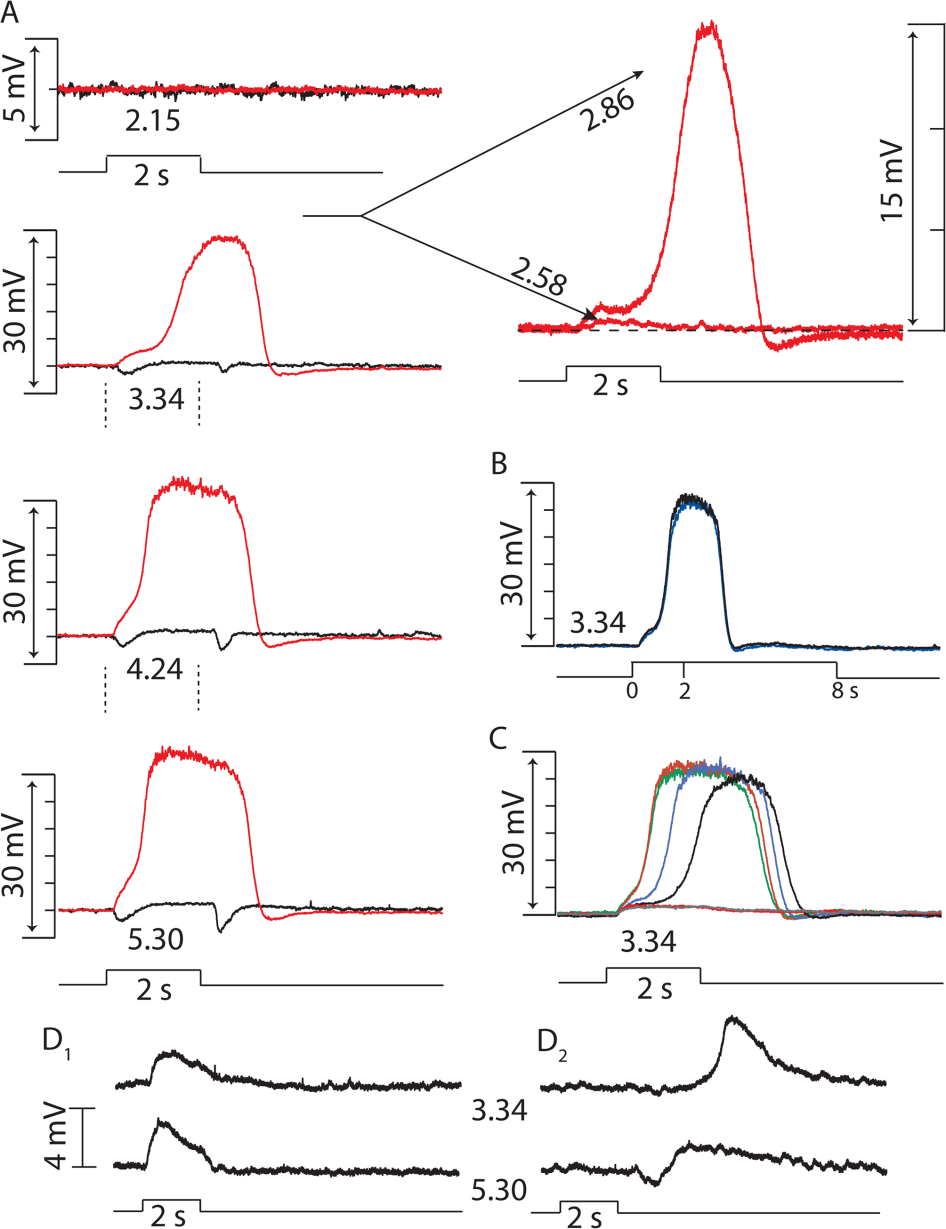
Effects of combinations of excitatory and inhibitory synaptic blockers on TNAC's response to light. (A) Light responses evoked by 2s flash of light at indicated intensities (Log10 Rh*/rod/s) before (black, V_rest_ -58 mV) and after (red) addition of 50 μM GABAzine (V_rest_ -53 mv) to bath solution containing 50 μM NBQX. (B) Light response evoked by 2 and 8s steps of light in presence of NBQX and GABAzine. (C) Superimposed light responses recorded before (red), and 2 (green), 5.25 (blue), 6 (black), 6.75 (gray), and 9.5 min (red) after adding 50 μM AP5 to bath solution containing NBQX and GABAzine. (D) Responses evoked by 2s light flashes at indicated intensities in (different cell, V_rest_ - 51 mV) in the presence of NBQX (50 μM), GABAzine (50 μM), TPMPA (50 μM) strychnine (10 μM) and AP5 (50 μM) before (D_1_) and after (D_2_) the addition of 62.5 μM L-APB.

The addition of NBQX to the AMES bath solution at a concentration (50 μM) that has been reported to block both AMPA and kainate type ionotrophic glutamate receptors [27,28,29,30] caused a small shift in resting potential that ranged from - 1 to + 4.5 mV in different cells (mean 1.14 +/- 0.85 mV, n = 6) and fully eliminated responses to dim light (< 300 Rh*/rod/s) while stronger photopic stimuli evoked small (2 to 6 mV) hyperpolarizing responses at light ON- and OFF-set (Fig 4). When GABAzine (either alone or in combination with TPMPA and strychnine) was added to bath solution containing NBQX photopic stimuli evoked a small slow depolarization at light onset that grew with increasing intensity and merged with a large (20 to 30 mV) long lasting (~ 2s) depolarization that recovered to resting potential with little overshoot (Fig 5A). The time course of the response was independent of the duration of the light step, indicating that its termination was not triggered by light offset (Fig 5B). The large depolarizing phase of the response was eliminated by AP-5 (Fig 5C), an NMDA receptor blocker, which had no effect on TNAC's resting potential or its response to light when added to the bathing solution by itself (data not shown).

**Fig 6:**
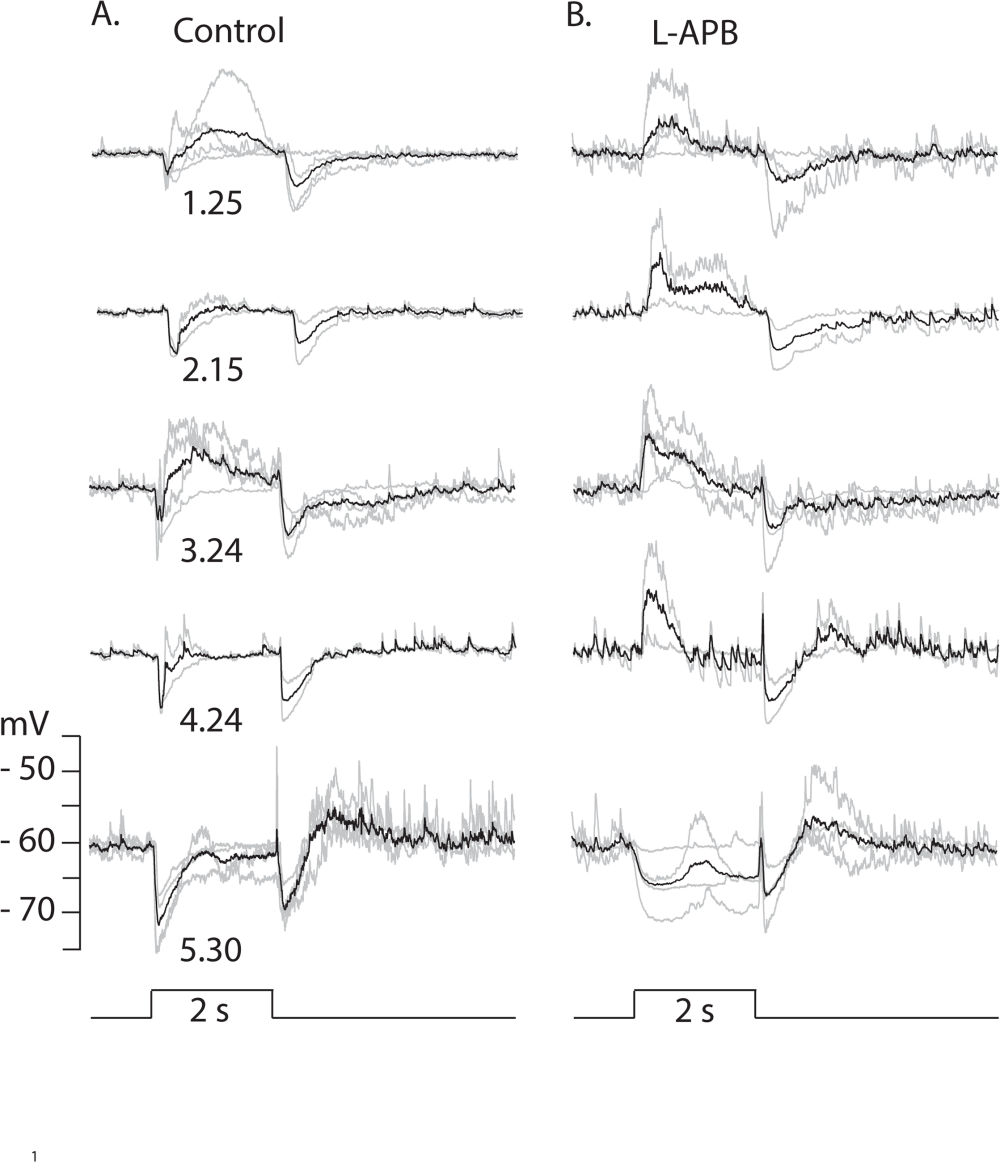
Effect of L-APB on TNAC responses to a range of light intensities. Right and left columns of traces show responses evoked by 2s flashes of full field 440nm light at the indicated intensities (log_10_ Rh*/rod/s) in the absence (A.) and presence (B.) of 62.5 μM L-APB. Each panel shows the individual responses recorded from n different cells (gray traces) and the their average (black). Resting potentials ranged across cells from -56 to -64 mV (mean -61 +/- 2.11, n = 5) and were set to - 60 mV for comparisons. The number of cells included in the average response at each intensity were: 1.25, n = 4; 2.15, n = 2; 3.34, n = 4; 4.24, n = 2; 5.40, n = 4.

The metabotropic glutamate receptor agonist (L-APB), which prevents ON type rod and cone bipolar cells from responding to light [31], blocked the initial hyperpolarizing response at light onset giving rise to a depolarizing response with a delayed recovery to either the resting dark potential or a small depolarized plateau and an inhibitory (hyperpolarizing) response at light off (Fig 6).

The small depolarization at light onset that remained in the presence of mixture of excitatory and inhibitory blockers, as described above (Fig 5C), was eliminated by L-APB, which also unmasked a delayed depolarizing OFF response that with stronger stimuli was preceded by a hyperpolarizing potential change (Fig 5D). While the light responses that remain in bathing solution containing this mixture of excitatory and inhibitory blockers are small in absolute size (≤ 5 mV) they are sizable in relation to the dynamic range of cell's responses to light in the absence of blockers (Fig 3).

#### Effects of perturbing inhibitory transmission on TNAC light response

**Fig 7:**
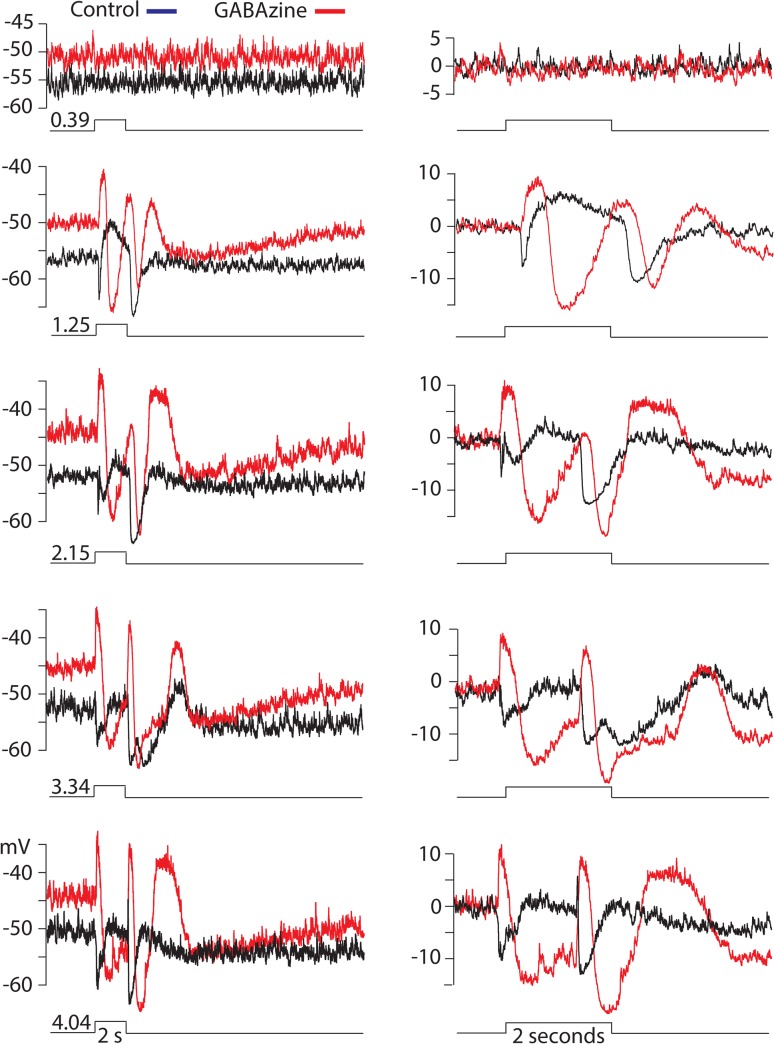
Effect of GABAzine on TNAC responses to a range of light intensities. Right and left columns of traces show responses evoked by 2s flashes of full field 440nm light at the indicated intensities (log_10_ Rh*/rod/s) on two different time scales in the absence (black) and presence (red) of 50 μM GABAzine. Panels show either single responses or an average of n responses. The number of responses each intensity were: 0.39, n = 2; 1.25, n = 5; 2.15, n = 1; 3.34, n = 1; 4.24, n = 2.

Strychnine had no effect on TNAC dark resting potential or its light response, whereas GABAzine, either alone or when combined with TPMPA and strychnine, caused dramatic changes in the response (Fig 7). The initial hyperpolarizing phase of the response at light onset was replacing with a large (10 to 20 mV) depolarizing potential change that recovered after a 300–600 ms delay to overshoot the resting dark potential by 10 to 15 mV before returning to a small (< 5 mV) depolarized plateau. Light offset evoked a strong hyperpolarizing response that recovered over the course of ~ 1s to the level of the depolarized plateau followed by a slower hyperpolarizing potential change that peaked approximately 2–3 s after the light had turned off (Fig 7).

**Fig 8:**
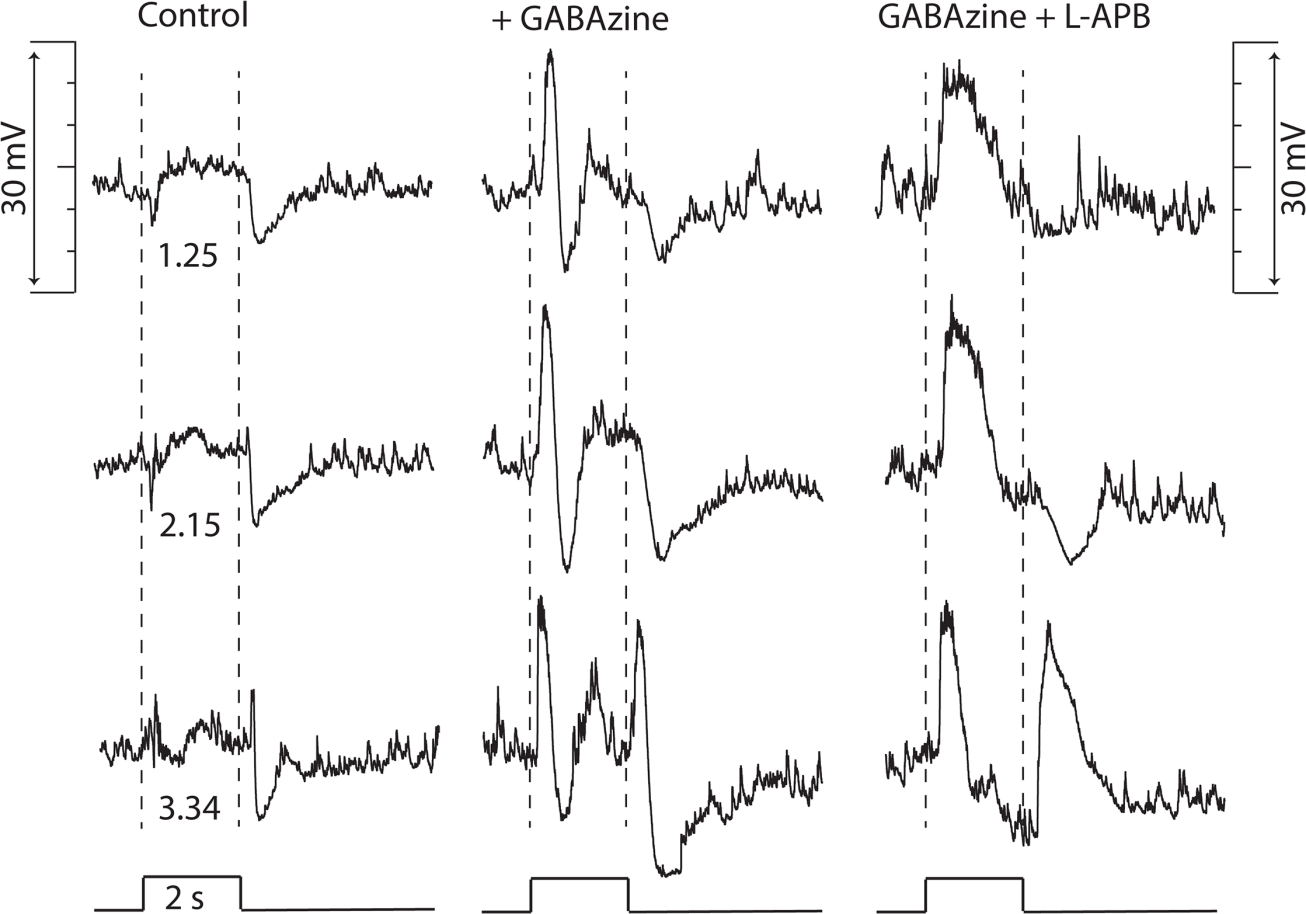
TNAC light responses persist in presence of GABAzine and L-APB. Traces in columns show responses evoked by 2s flashes of full field 440nm light at the indicated intensities (log_10_ Rh*/rod/s) in Ames solution (left), after addition of 50 μM GABAzine (middle) and after addition of 75 μM L-APB (right). The resting potential was - 64 mV in control and - 59 mV and - 60 mV in GABAzine and GABAzine + L-APB, respectively.

In the presence of GABAzine the addition of L-APB increased the duration of the depolarizing potential change and eliminated the recovery phase that over shot resting dark potential with a small reduction of the hyperpolarizing response at light offset and elimination of the slow delayed hyperpolarizing phase of the GABAzine response (Fig 8).

## Discussion

Our experiments have documented the basic properties of a tristratified narrow-field amacrine cell (TNAC) that we equate with amacrine cell 42 (AC42) in Helmstaedter et al.'s connectomic study of the mouse inner plexiform layer [25]. The correspondence between TNAC and AC42 is based on the similarity of the radial extent and location of the three separate dendritic ramifications and the size and position of the soma in the INL. None of the other morphological surveys of amacrine cells include a cell with a similar set of morphological features [1,17,32,33].

AC42's third dendritic ramification is apparent in Helmstaedter's supplemental data figure (Nature 12346-s2.pdf, page 42) as a broad increase in mean skeleton density in the vicinity of 80% IPL depth. While this increase in neurite density is real, it is admittedly small especially when compared to the increase in fluorescence intensity that distinguishes TNAC's third arborization in the same region of the IPL (Fig 1E). In comparing the strength of the neurite signals in Helmstaedter's and our Z-maps of the IPL, it is important to note that Helmstaedter's process tracings are skeletons, which assume a fixed dendrite diameter and represent a linear density. The fluorescence map plots a volumetric density that is augmented by including fluorescence from dendritic varicosities and lobules, which the skeleton map does not take into account. Since the neurites that make up TNAC's inner most arborization are lobular and beaded with varicosities, one would expect that their prominence would be under estimated by a map based on skeleton density. The morphological similarities between AC42 and TNAC are sufficient to justify the following speculations about how AC42's connectomic data might be used to inform hypotheses about TNAC's synaptic circuitry and function.

Helmstaedter's analysis [25] of cell-to-cell contacts indicates that 37% of the total TNAC contact area is occupied by bipolar cell terminals. Of the contacts with bipolar cells 42% of the area is with OFF bipolars, mainly cone bipolar cell (CBC) type 3 and type 4 (25 and 13%, respectively). The remaining 58% of TNAC's contact area with bipolar cells is with ON bipolar cells (70% cone and 30% rod ON bipolar cells). Of the cone ON bipolar cell contacts most (72%) is contributed by CBC type 5, which ramifies in the middle (~ 50%) of the IPL, where it co-stratifies with the newly identified [25] X type bipolar cell (XBC); the second largest contributor (18%) to ON cone contact area on TNAC.

Based on optical recordings of Ca2+ signals in identified bipolar cell axon terminals [34] the type 5 CBC is unique among the CBCs in that it responds with solely a transient increase in Ca2+ at the ON- and OFF-set of a 4s step of light, with each transient increase recovering in ~ 500ms to a Ca2+ level that is the same as the level in darkness. In the terminals of OFF type 3 and 4 CBCs there is a sustained decrease in Ca2+ during light exposure that transiently increases at light OFF-set before returning to baseline dark level. The transient increase in Ca2+ at light off in the type 4 CBC is similar in amplitude and kinetics to the transient changes in Ca2+ in CBC type 5, consistent with the reported propensity of bipolar cells terminating in middle of the IPL to support the generation of transient as opposed to sustained responses to light [34,35].

Information about differences in the temporal response properties of identified bipolar cells based on either intracellular recording [36] or optical recording in which increases and decreases in Ca2+-dependent fluorescence are interpreted as depolarizing and hyperpolarizing changes in membrane voltage (respectively) suggests that the excitatory input to TNAC is predominately transitory at light onset and offset; 76% of the bipolar contact area is with bipolar cells that respond at light ON- and/or OFF-set with a transient depolarization. This is in comparison to 20% of the contact area being from bipolar cells that respond to light with a sustained depolarization (RBC, CBCs 7,8 & 9) or hyperpolarization 4% (CBC 1,2,3,& 4). While the synaptic directionality of the bipolar amacrine contacts is not known (the amacrine cell could be either the post or presynaptic element) the predominance of contacts with transient bipolar cell types is consistent with the transient properties of ON and OFF components of TNAC light response (Fig 3).

Forty eight percent of the total contact area of AC42 is with amacrine cells [25]. While the synaptic direction of these contacts is unknown, it is clear from the neuropharmacology that that TNAC does not receive glycinergic input and thus it might be expected that in most cases TNAC is the presynaptic element at its contacts with narrow field ACs since these are commonly assumed to be glycinergic [37]. It is not, however, possible to use the Helmstaedter's connectomic data to cleanly segregate the 45 different amacrine cell types in their 114 μm by 80 μm section of retina into narrow, medium and wide field cells in the conventional sense of these categories since amacrine cells with dendritic arbor diameters less than 125 μm are typically classified as narrow field [33]. In which case any AC with an arbor fully enclosed in Helmstaedter's sample [25] would be considered narrow field cell while the classification of amacrine cells with dendritic processes that exceeded the boundaries of the connectomic section would be indeterminate.

Of the identified ACs it appears that TNAC provides input to the glycinergic AII amacrine cell, which accounts for 6% of its total AC contact area. The largest fraction (8.2%) of its contact area is with A17 amacrine cells, which makes the A17 a strong candidate for being one of the sources of GABAergic input to TNAC. The second largest fraction of TNAC's AC contact area (6.2%) is with other TNACs, which likely represent gap junctional contacts consistent with TNACs being electrical coupled (dye coupled) to each other (Fig 1C).

### Neural Circuitry: A Proposal

**Fig 9:**
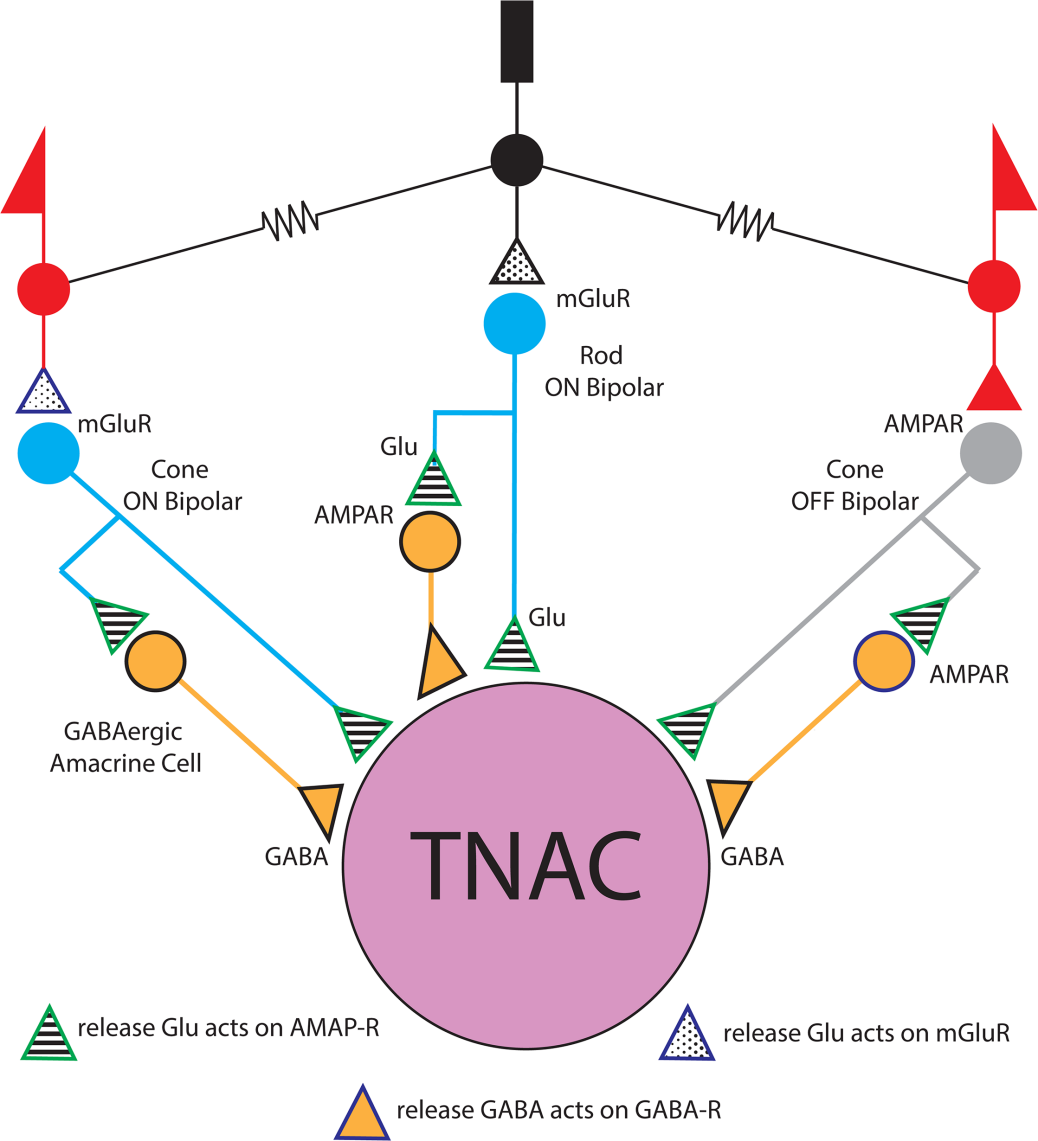
Potential TNAC synaptic circuitry. TNAC presumably receives direct excitatory (glutamatergic) input from rod and cone ON and OFF bipolar cells and inhibitory input from unidentified GABAergic amacrine cells that are driven by the rod and cone ON and OFF bipolar cells. The correspondence between TNAC and AC42 in Helmstaedter et al.'s connectomic analysis (2013) of the mouse retina suggests that the predominate sources of ON and OFF cone bipolar cell (CBC) input is CBC type 5 and CBC types 3 & 4, respectively. The circuit diagram does not show the network of gap junctional coupling between TNACs or between TNAC and an unidentified retinal neuron in the inner nuclear layer (see Fig 1).

We consider a circuit in which TNAC receives input from three similar modules, one driven by rods and two by cone photoreceptors (Fig 9). Two of the modules are based on ON type bipolars (one driven by input from rods and the other from cones) and third is based on OFF type CBCs. The connectomic data taken at face value would suggest that the cone driven modules are comprised principally of CBC5 and XBC for the ON pathway and CBC 3 and 4 for the OFF pathway. The bipolar cell type in each of the three modules also makes excitatory contact with GABAergic amacrine cells that provides inhibitory input to TNAC (Fig 9). The proposed circuit can account qualitatively for some but not all of our results.

The spontaneous voltage fluctuations in darkness were eliminated by NBQX without a concomitant change in resting potential whereas GABAzine had no effect on the dark noise but caused a depolarizing shift in resting potential (Fig 2). Neither strychnine nor L-APB had any effect of the noise or the resting membrane potential. These results are consistent with the circuit in Fig 9 and support the conclusion that TNACs receive steady glutamatergic and GABAergic input in darkness. NBQX would block glutamate receptors on TNAC that give rise to the dark noise and on presynaptic GABAergic amacrine cells that are activated by glutamatergic input from OFF CBCs in darkness. Under these conditions the elimination of both the excitatory and inhibitory input by NBQX could have offsetting effects in a way that results in no net change in resting potential. GABAzine on the other hand eliminates only the inhibitory input to TNAC causing a decrease in the resting potential without a change in baseline voltage noise, which might be expected to be dominated by fluctuations in excitatory rather than inhibitory currents because of the difference in driving force (E_rev_^Glu^—V_rest_ >> E_rev_^GABA^—V_rest_). The source of the continuous glutamatergic input that causes the noise in darkness could be the terminals of either ON or OFF bipolar cells. OFF bipolar cells are depolarized in darkness and presumably release glutamate continuously. Although ON bipolar cells are hyperpolarized in dark, fluctuations in their resting potential could trigger the discontinuous release of glutamate and contribute to the generation of synaptic noise in TNAC. L-APB, an mGluR6 agonist, blocks the depolarizing light response of ON bipolar cells by locking-in the hyperpolarized resting dark state and thus would not be expected to change the characteristics of stochastically released glutamate and have no effect on TNAC dark noise if ON bipolar cell input contributed to its generation.

TNAC's threshold light response is a transient hyperpolarization at light ON and OFF (Fig 3). Scotopic stimuli excite rods causing a sustained depolarization in RBCs [36] and presumably a maintained increase in glutamate release at their synaptic contacts on TNAC and GABAergic ACs in the proposed circuit (Fig 9). This would increase both excitatory and inhibitory input at light ON and decrease both at light OFF. It is possible that the temporal properties of the amacrine cell response and/or difference in the kinetics of transmitter release at the intervening synapses are such that the increase in inhibition predominates at light ON and the decrease in excitation predominates at light off giving rise to a hyperpolarizing OFF response. The generation of TNAC's threshold response might also involve cone pathways activated by scotopic stimuli via rod-cone electrical coupling. In the proposed circuit the participation of cone-driven pathways could have number of potential consequences. In the ON pathway the excitation of the ON/OFF response of CBC type 5 would be expected to give rise to a transient increase in the release of glutamate and GABA at light ON- and OFF-set. In the cone-driven OFF pathway the expectation would be a decrease in both excitatory and inhibitory input at light ON and increase in both at light OFF.

It is clear that the three different potential sources of excitatory and inhibitory synaptic input in the proposed circuit could be summed to give rise to either transient hyperpolarizing or depolarizing responses at light ON- and OFF-set depending their weight and kinetics. The variables that control these parameters and ultimately determine the waveform of the light response are not known, which illustrates the impossibility of understanding retina function of the basis of circuitry alone.

The results of the neuropharmacology experiments increase further the number of uncertainties in the proposed circuit by showing that conventional assumptions about the synaptic mechanisms do not apply. These inconsistences were as follows:

1. NBQX abolishes responses evoked by dim but not bright light (Fig 4). In the presence of the blocker ON- and OFF-set of a photopic stimulus produced a brief hyperpolarizing potential change. The elimination of the scotopic response is consistent with NBQX blocking excitatory input to TNAC and to GABAergic amacrine cells in the ON and OFF pathways. The persistence of the response to photopic stimuli in the presence of NBQX, with or without the addition of UB310, a selective Kainate receptor blocker (data not shown), can not be explained on the basis of conventional assumptions about the neuropharmacology of the synaptic elements in Fig 9.
2. Photopic stimuli in the presence of NBQX and GABAzine evoked a large (15 to 20 mV) long duration (~2s) depolarizing response that recovered with a waveform that was independent of the length of the stimulus and didn't include an OFF response (Fig 5). The large depolarizing response was preceded by a small depolarizing pre-potential that got larger and faster with increasing light intensity. AP-5, an NMDAR antagonist, blocked the large depolarizing phase of the response but not the depolarizing pre-potential. The changes in the photopic light response were consistently observed in all experiments done in the presence of NBQX and GABAzine but we are unable explain them on the basis of the circuit in Fig 9 using the common presumptions about the identity of the neurotransmitters and their synaptic interactions. This point is reinforced by the persistence of a photopic light response in presence of a mixture of NBQX, L-APB, AP-5, GABAzine, TPMPA, and strychnine (Fig 5D).
3. We are also at a loss to explain the results obtained with L-APB. In the presence of the mGluR6 agonist the response to dim as well as bright light is depolarizing at the ON- and hyperpolarizing at OFF-set of light (Fig 6). The OFF pathway in Fig 9 could generate a depolarization at light ON-set by suppressing excitatory input to the GABAergic AC thereby reducing the inhibitory input to TNAC (dis-inhibition). At light OFF-set the reversal of this chain of events could account for the hyperpolarizing OFF response. This hypothesis is not, however, compatible with the persistence of the ON / OFF response in the presence of L-APB and GABAzine (Fig 8) with or without the inclusion of TPMPA and strychnine. The possibility that responses under these conditions are driven by intrinsically photosensitive retinal ganglion cells (IPRGs) is inconsistent with the failure of scotopic stimuli to elicit melanopsin triggered responses in IPRGs [38]. In the absence of the ON pathway and inhibitory input to TNAC the generation of a depolarizing response might be explained by the involvement of GABAergic amacrine cells that release a second neurotransmitter that acts through 2nd message enzyme cascade to influence intrinsic conductances [39]. The A17 amacrine cell might be such a cell, it is GABAergic, accumulates indolamine [8,40] and accounts for the largest fraction of TNAC's contact area with amacrine cells [25].

Speculation based on unknown transmitters acting via unknown signal transduction pathways to influence unknown intrinsic conductances in not satisfying since such an explanation could in principle account for the effect of nearly any pharmacological intervention. But the fact is the results of our experiments using canonical neuropharmacology were consistent from one cell to the next yet could not be understood on the basis of conventional thinking about synaptic interactions in retina circuits. Information about the anatomy of the circuitry is useful but not nearly enough to understand how the retina works.

### TNAC Function

The basic visual computation performed by TNAC is differentiation. It signals increases and decreases in luminance with a transient hyperpolarization that would presumably reduce inhibitory input to its down stream synaptic targets. In this way TNACs may enhance the effects of excitatory input to ganglion cells that fire a transient burst of spikes when light is turned on and when it is turned off. According to Helmstaedter's analysis ganglion cells make up 15% of the TNAC's contact area and of this the largest fraction (25%) is with W3 ganglion cells; a local motion detector in the mouse retina with an ON-OFF receptive field [41]. This raises the possibility that TNAC mediated disinhibition plays a role in the neural circuitry that gives rise to edge detection in a select set of ganglion cell types [42,43]. Hypotheses about TNAC's role in visual behavior could be evaluated by using genetics to silence these cells.
